# Metastatic Merkel Cell Carcinoma Incidentally Detected on PSMA PET/CT in a Patient With Metastatic Prostate Cancer

**DOI:** 10.1155/crra/1751973

**Published:** 2025-10-31

**Authors:** William Y. Raynor, Manas Ranpariya, Jeffrey S. Kempf, Biren Saraiya, Sarah A. Weiss

**Affiliations:** ^1^Department of Radiology, Rutgers Robert Wood Johnson Medical School, New Brunswick, New Jersey, USA; ^2^Rutgers Robert Wood Johnson Medical School, New Brunswick, New Jersey, USA; ^3^Division of Medical Oncology, Department of Medicine, Rutgers Cancer Institute, New Brunswick, New Jersey, USA

**Keywords:** ^18^F-DCFPyL, FDG, MCC, neuroendocrine, positron emission tomography, PSA

## Abstract

Positron emission tomography (PET) radiotracers targeting prostate-specific membrane antigen (PSMA) are now widely used in the evaluation of prostate cancer. However, PSMA activity has also been described in several nonprostatic malignancies, where PSMA is primarily expressed in tumor neovasculature. Here, we describe to the best of our knowledge the first case of a PSMA-avid Merkel cell carcinoma (MCC) inguinal lymph node metastasis, detected incidentally in an 80-year-old man with advanced metastatic prostate adenocarcinoma. Clinical history and disease distribution prompted the need for a diagnostic biopsy, confirming PSMA-avid metastatic MCC. This case highlights the importance of recognizing nonprostatic causes of PSMA uptake, as synchronous malignancies can alter diagnostic interpretation and treatment planning.

## 1. Introduction

Molecular imaging with prostate-specific membrane antigen (PSMA) positron emission tomography/computed tomography (PET/CT) is currently used in the staging of prostate cancer, for the assessment of biochemical recurrence after therapy, as well as for planning for radiopharmaceutical treatment targeting PSMA. The radiotracers used in PSMA PET (including ^18^F-DCFPyL, ^68^Ga-PMSA-11, and ^18^F-rhPSMA-7.3) bind to PSMA, which is a transmembrane protein highly expressed in prostate cancer cells, allowing for detection of prostate cancer metastases at a sensitivity and specificity rate of 73.7% and 97.5%, respectively [1]. PSMA PET/CT is not only useful for detecting and staging prostate cancer but also increasingly plays a role in guiding prostate biopsy for the diagnosis of clinically significant prostate cancer. Recent data also demonstrate the value of SUVmax in identifying high-grade lesions [2]. Beyond the established utility in prostate cancer, PSMA PET/CT has demonstrated potential to detect other malignancies, such as renal cell carcinoma (RCC), due to the expression of PSMA in tumor neovasculature [3]. A lesion-based analysis of five patients with clear cell RCC found a sensitivity of 95% by PSMA PET/CT, compared to 79% by conventional imaging (CT and MRI) [4]. This broader diagnostic capability can occasionally lead to the incidental discovery of other cancers. Here, we report the first known case, to the best of our knowledge, of a patient with metastatic prostate adenocarcinoma in which PSMA PET/CT facilitated the incidental discovery of a Merkel cell carcinoma (MCC) lymph node metastasis.

## 2. Case Presentation

An 80-year-old man with a history of metastatic prostate cancer diagnosed 7 years prior presented to the dermatologist with an atypical cutaneous lesion in the left anterior pretibial region. Clinically, this lesion appeared as a pearly telangiectatic papule [5]. Biopsy was performed, and pathology showed neuroendocrine carcinoma positive for pancytokeratin and CK20 with a paranuclear dot staining pattern consistent with MCC. Wide local excision was performed. The MCC was 1 cm in diameter and 5 mm in depth with lymphovascular invasion and a mitotic rate of 14/high power field. Surgical margins were negative. No sentinel lymph node biopsy was performed due to the presence of medical comorbidities including advanced prostate cancer for which the patient was receiving active treatment, and thus, close surveillance was planned.

**Figure 1:**
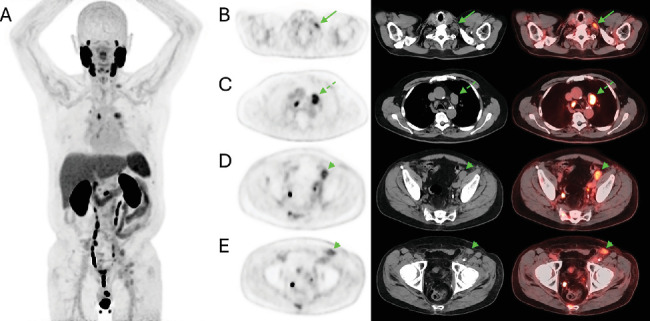
Maximum intensity projection (MIP) from a PSMA PET/CT study (A) performed for restaging of prostate cancer. Multiple new sites of PSMA-avid disease were detected including a left supraclavicular lymph node (*arrow*, B) measuring 19 × 13 mm with SUVmax 4.0. Mediastinal lymphadenopathy was present, including a subaortic lymph node (*dotted arrow*, C) measuring 33 × 21 mm with SUVmax 6.3. In addition, PSMA activity was found to localize to a left external iliac lymph node (*arrowhead*, D) measuring 25 × 17 mm with SUVmax 4.4. A left inguinal lymph node (*arrowhead*, E), later found to represent biopsy-proven metastatic MCC, measured 22 × 14 mm with SUVmax 3.4.

He was diagnosed with Gleason 7 (3 + 4) prostate adenocarcinoma 7 years prior and underwent prostatectomy and adjuvant radiation, after which prostate-specific antigen (PSA) was undetectable (< 0.01 ng/mL). Osseous metastases were detected on bone scintigraphy 3 years later, at which point PSA measured 0.53 ng/mL, and he was initially treated with enzalutamide, androgen deprivation therapy, and denosumab and subsequently enrolled in a clinical trial. Due to clinical decline and concern for progression of metastatic prostate cancer, PSMA PET/CT was performed 18 days after the MCC wide excision at which time PSA measured 1.8 ng/mL. In addition to the known metastatic osseous disease such as in the L2 vertebral body (SUVmax 10.1), ^18^F-DCFPyL PET/CT (Figure 1) revealed new PSMA-avid mediastinal (SUVmax 6.3) and left supraclavicular (SUVmax 4.0) adenopathy suspected to represent prostate cancer metastases. In addition, increased PSMA activity was observed corresponding to borderline enlarged left iliac (SUVmax 4.4) and left inguinal (SUVmax 3.4) lymph nodes, although the degree of uptake was notably less than that of the metastatic sites in the chest and axial skeleton.

**Figure 2:**
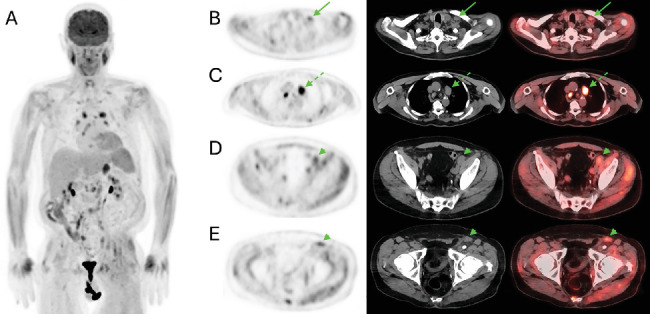
FDG PET/CT MIP (A) performed for staging of MCC approximately 1 month after the PSMA PET/CT shown in Figure 1. Increased FDG uptake was associated with the left supraclavicular lymph node (SUVmax 2.8, *arrow*, B), left external iliac lymph node (SUVmax 3.2, *arrowhead*, D), and left inguinal lymph node (SUVmax 3.3, *arrowhead*, E), with higher FDG uptake demonstrated by the subaortic lymph node (SUVmax 7.4, *dotted arrow*, C).

At his postoperative visit 8 days after the MCC excision, newly palpable left inguinal adenopathy was discovered on physical examination, and ultrasound performed 4 days later confirmed enlarged lymph nodes, corresponding to the findings on PSMA PET/CT. An ultrasound-guided biopsy was completed 9 days after the PSMA PET/CT was performed. Pathology showed neuroendocrine cells consistent with MCC, positive for CK20 and negative for PSA, confirming that the PSMA-avid left inguinal lymphadenopathy represented metastatic MCC rather than prostate cancer. ^18^F-fluorodeoxyglucose (FDG) PET/CT performed for MCC staging showed elevated FDG uptake (SUVmax 3.3), which was higher than that of the hepatic parenchyma (SUVmean 2.0), corresponding to the previously noted PSMA-avid left inguinal lymph nodes (Figure 2).

Anti-PD-1 immunotherapy with pembrolizumab was initiated for the treatment of MCC [6]. After three cycles of pembrolizumab, he developed immune-mediated arthritis requiring steroids. Follow-up FDG PET/CT performed 3 months after the initiation of pembrolizumab showed a mixed response to therapy, while PSA rose to 4.8 ng/mL. Specifically, there was progression of the left inguinal adenopathy, with SUVmax increasing from 3.3 to 7.1 with a relatively stable appearance on CT. Meanwhile, the left supraclavicular lymphadenopathy had essentially resolved, while multiple mediastinal lymph nodes and bone metastases increased in size and activity. Due to a decline in his performance status and the progression of his disease, the patient was ultimately referred to hospice care.

## 3. Discussion

Upon review of the medical literature, this is the first documented case of PSMA-avid MCC that we have found using PSMA PET/CT. Adding to the number of nonprostatic PSMA-avid diseases [7–10], this case emphasizes the need for careful consideration for synchronous pathology when PSMA PET is performed for prostate cancer staging. Typical patterns of disease spread can be useful in the distinction between prostatic and nonprostatic causes of PSMA uptake, and while isolated inguinal lymph node metastases from prostate cancer are rare, prostate cancer metastasizing to inguinal lymph nodes is not impossible [11]. The key insight leading to the diagnosis of PSMA-avid MCC inguinal lymph node metastasis in this case was clinical correlation with the patient's known recent left lower extremity MCC excision, prompting the need for biopsy of the atypical left inguinal lymph nodes noted on PSMA PET/CT. Although uncommon, the correct identification of PSMA uptake due to causes other than prostate cancer is critical to inform treatment plan decisions. A low threshold for biopsy is warranted in suspected cases, particularly if there is a history of a second malignancy or if the pattern of metastatic spread is questionable or atypical.

MCC is a rare and aggressive skin cancer, so named given the resemblance between the tumor cells and the normal Merkel cells found in the basal layer of the epidermis [12], although more recent data suggest that MCC more likely arises from an epithelial (follicular) progenitor cell [13]. Both Merkel cells and the tumor cells found in MCC express neuroendocrine markers, including chromogranin-A [14, 15]. Exposure to ultraviolet radiation and Merkel cell polyomavirus are implicated in most cases of MCC [16], which often presents as a cutaneous or subcutaneous nodule. A meta-analysis of 721 patients with MCC determined that locoregional metastases were present at diagnosis in approximately 30% of cases [17]. Wide excision of the primary and sentinel lymph node biopsy and/or adjuvant radiation therapy serve as first-line management, while anti-PD-1 immune-checkpoint inhibition plays a role in locally advanced (Stage III) and metastatic (Stage IV) disease [18].

The optimal method for MCC staging is still uncertain, although the current evidence favors the use of FDG PET/CT, which identifies sites of disease based on elevated glucose metabolism by tumor cells [19]. Several studies show high sensitivity and specificity of FDG PET/CT in detecting MCC metastases, often resulting in patient upstaging [20–24]. A 2022 meta-analysis which examined 259 patients across nine studies by Shim et al. found a 91% sensitivity and a 93% specificity for nodal staging of MCC by FDG PET/CT [25]. By comparison, CT alone may be less sensitive for nodal staging of MCC, with Colgan et al. reporting a sensitivity of 47% [26]. Similarly, Hawryluk et al. reported FDG-avid bone and bone marrow MCC metastases in 10 patients which did not have appreciable abnormalities on CT [24]. Limitations of FDG PET/CT include low uptake in well-differentiated tumors and also false positive uptake from sites of infection or inflammation [27]. Given the expression of somatostatin receptors (SSTRs) by tumor cells in MCC, SSTR PET can also be considered, with tracers such as ^68^Ga-DOTATATE and ^68^Ga-DOTATOC binding to the SSTR2 subtype with particularly high affinity [28]. A recent review of the current evidence recommended FDG PET/CT for initial MCC staging, while SSTR PET/CT may be useful if FDG PET/CT is negative and clinical suspicion for metastatic disease remains high [29].

PSMA PET is now widely used for detecting and staging prostate cancer by identifying cells that express the PSMA protein. While PSMA is expressed to a certain extent by normal epithelial prostate cells, there is significant overexpression by tumor cells in prostate cancer, with more aggressive disease generally expressing higher levels of PSMA [30]. Despite the overall high sensitivity of PSMA PET/CT for prostate adenocarcinoma, limitations exist in certain histologic variants, with a case report describing false negative staging of ductal prostate carcinoma in two patients [31]. At the same time, PSMA uptake has been found to be associated with an increasing number of nonprostatic conditions (including infectious/inflammatory processes such as sarcoidosis, benign neoplasms such as meningiomas, and malignancies such as high-grade gliomas), indicating that the marker is not as specific for prostate cancer as once believed [7, 8]. Recognizing PSMA uptake due to causes other than prostate cancer is critical to avoid misdiagnosis. While nonprostatic PSMA uptake hampers accuracy in prostate cancer staging, it also expands potential applications of PSMA PET, which as a result has been proposed for staging and restaging of other malignancies such as RCC [32, 33].

Use of PSMA PET has been reported in some tumors with neuroendocrine characteristics, notably neuroendocrine prostate cancer (NEPC), which is a rarer and more aggressive subtype of prostate cancer compared to prostate adenocarcinoma. NEPC can arise de novo as small-cell carcinoma of the prostate in a small proportion of cases but more often results from adenocarcinoma transformation as a form of treatment resistance [34]. NEPC typically exhibits reduced PSMA expression, with a corresponding increase in glucose uptake and SSTR expression, making FDG and SSTR PET/CT potentially viable methods of detection [35]. However, cases of intensely PSMA-avid NEPC have been reported [36], and the use of PSMA PET in neuroendocrine transdifferentiation of prostate cancer remains an area of ongoing investigation. In prostate cancer, PSMA is primarily expressed on the apical membrane, leading to strong tracer uptake, while nonprostatic tumors typically have cytoplasmic PSMA expression, resulting in low tracer localization [37, 38]. Rather, PSMA expressed in the endothelium of the tumor-associated neovasculature is thought to be responsible for uptake observed in nonprostatic PSMA-avid tumors [39]. This is suspected to be true for MCC as well, corroborated by a study by Ramirez-Fort et al., in which immunohistochemistry performed on 81 primary and metastatic MCC lesions showed prevalent PSMA expression associated with the tumor neovasculature in 67% (54/81) of patients [40]. Immunohistochemical testing for PSMA expression was not performed in the present case, but future studies could consider it to evaluate this hypothesis. While no quantitative threshold exists to discriminate between causes of PSMA uptake [41], the relatively mild PSMA uptake demonstrated by metastatic MCC compared to the higher PSMA uptake demonstrated by prostate cancer metastases we observed is concordant with the typical patterns of nonprostatic uptake others have reported [8, 37].

## 4. Conclusion

Although the tumor biology of MCC is more suitable for detection by FDG or SSTR PET, the recognition of PSMA uptake by metastatic MCC represents an important observation for patients with synchronous MCC and prostate cancer. Including MCC or potentially other tumor types in the differential diagnosis for PSMA-avid lesions in patients with the appropriate clinical history and disease distribution could influence the decision to biopsy indeterminate lesions, potentially changing the course of treatment.

## Data Availability

Data sharing is not applicable to this article as no datasets were generated or analyzed during the current study.
